# The Recent Meeting of the American International Congress on Tuberculosis in New York

**Published:** 1906-12

**Authors:** 


### EDITORIAL DEPARTMENT.

### THE RECENT MEETING OF THE AMERICAN INTERNATIONAL CONGRESS ON TUBERCULOSIS IN NEW YORK.

As per announcement a three-days' session of the American International Congress on Tuberculosis was held at Hotel Astor in New York November 14, 15, and 16. Though the attendance was not so large as was expected, it was fairly representative of many nations. Papers were read and addresses and discussions delivered in English, German, French, Spanish and Italian. Many notable papers were read, amongst which I recall one by Dr. T. D. Crothers, of Hartford, on Tuberculosis and Alcohol; one by Professor D'Olise, of Naples, Italy; one by the delegate from Hayti, Professor Jeanti; one by Von Schoen, of Germany; one by Dr. C. M. Amende, of New York, advocating a "Return to Nature" as the best preventive and cure for tuberculosis. The President's address was entitled, "The Duty of the Government to Its People" (published herewith). This address has been widely (mis)quoted throughout the United States and much commented upon by the leading dailies everywhere. Resolutions were reported by the Committee on Resolutions and unanimously adopted, in accordance with the President's suggestions, that a Department of Public Health with a minister in the President's cabinet is imperatively demanded by every consideration of public policy, economy and in the name of humanity.

A dinner by the Medico-Legal Society of New York, complimentary to the Congress, was given at the Hotel Astor on the evening of November 15. Hon. Clark Bell, President of the Medico-Legal Society, presided as toast-master. Toasts were drunk and responded to by the President of the Congress, Dr. Daniel, Judge Frank Ross, Judge Miller, Judge Francis and Judge Bell, all of the New York bar; Dr. M. M. Smith, Secretary of the Congress; Signor Ulloa, of Costa Rica; Professor D'Olise, of Naples; Colonel Bean, of El Paso; Mrs. Rorer, of *cuisine* fame; Mrs. Judge Cantrell, of Kentucky, and other notables. It is to be regretted that I took no notes and that all this eloquence will be lost to the world unless the indefatigable Clark Bell got it for the *Medico-Legal*

*Journal* and the *Bulletin*. Clark Bell, the ubiquitous, the indefatigable, the perennially youthful and debonair; the inimitable host and delightful friend, is a genius, the genius of organization and executive ability. He bore off all the honors. To him and him alone is due the gratifying success of the meeting. May his days be lengthened into many more years of usefulness and activity in the good cause of humanity.

Invitations to hold the next meeting were received from the exposition management at Jamestown, from Atlantic City, and from New Orleans. The new President is Dr. C. H. Irion, of New Orleans, President of the Louisiana State Board of Health; Dr. Geo. R. Tabor, State Health Officer of Texas, is First Vice-President; Dr. Drewry, of Petersburg, Va., Dr. Fassett, of Missouri, and Dr. Fest, of New Mexico, Vice-Presidents. Dr. M. M. Smith, of Austin, re-elected Secretary and made Treasurer, vice Clark Bell, retired.

